# Investigation of Content and Face Validity and Reliability of Sociocultural Attitude towards Appearance Questionnaire-3 (SATAQ-3) among Female Adolescents

**Published:** 2017-01

**Authors:** Somayeh Mousazadeh, Mahnaz Rakhshan, Fateme Mohammadi

**Affiliations:** 1 Ph.D Candidate in Nursing, Department of Nursing, Student Research Committee, Shiraz University of Medical Sciences, Shiraz, Iran.; 2 Assisstant Professor, Community Based Psychiatric Care Research Center, School of Nursing and Midwifery, Shiraz University of Medical Sciences, Shiraz, Iran.; 3 Ph.D Candidate in Nursing, Department of Nursing, Student Research Committee, Shiraz University of Medical Sciences, Shiraz, Iran.

**Keywords:** *Body Image*, *Female Adolescents*, *Psychometrics*, *Sociocultural Attitude towards Appearance*

## Abstract

**Objective: **This study aimed to determine the psychometric properties of sociocultural attitude towards appearance questionnaire in female adolescents.

**Method:** This was a methodological study. The English version of the questionnaire was translated into Persian, using forward-backward method. Then the face validity, content validity and reliability were checked. To ensure face validity, the questionnaire was given to 25 female adolescents, a psychologist and three nurses, who were required to evaluate the items with respect to problems, ambiguity, relativity, proper terms and grammar, and understandability. For content validity, 15 experts in psychology and nursing, who met the inclusion criteria, were required. They were asked to assess the qualitative of content validity. To determine the quantitative content validity, content validity index and content validity ratio were calculated. At the end, internal consistency of the items was assessed, using Cronbach’s alpha method.

**Results:** According to the expert judgments, content validity ratio was 0.81 and content validity index was 0.91. Besides, the reliability of the questionnaire was confirmed with Cronbach’s alpha = 0.91, and physical and developmental areas showed the highest reliability indices.

**Conclusion**: The aforementioned questionnaire could be used in researches to assess female adolescents’ self-concept. This can be a stepping-stone towards identification of problems and improvement of adolescents’ body image.

Body image is one of the main dimensions of self-concept (1) It affects individuals’ physical, emotional, social, and attitude understanding as well as various aspects of their psychological, social, sexual, familial, and adaptive identity (2). In fact, body image and positive attitude towards body have been identified as one of the most important criteria of mental health (3). On the other hand, body image has a multidimensional structure, but is normally defined as a degree of satisfaction with physical appearance (4). As physical appearance is a major part of an individual’s identity, and it is manifested through social occasions and behavior with others, it is of particular importance. 

Yet, the extent of attention to this personality construct is different in various sectors of the society (3, 5). 

Evidence has demonstrated that body image and physical attraction is more important to females compared to other society members (3). In the U.S., body image is a major concern among girls and women (6). This causes female adolescents to make great attempts to have a perfect appearance. However, in case they do not achieve their ideal appearance, they will develop negative emotions, eventually leading to stress, anxiety, and depression (7).

One of the main reasons for dissatisfaction with body image is inappropriate cultural atmosphere and social criteria ruling the community (1) since, even inappropriate, social and cultural criteria based on the accepted characteristics in the society affect formation of body image in individuals (8).

Mass media also play a critical role in spreading these criteria in the society, particularly in developing countries (3, 9).

The findings of the study by Knauss et al. (2007) in Switzerland revealed a significant relationship between satisfaction with body image and internalization of media-communicated ideals and encouragement by media to reach these ideals (10). Similarly, Jones et al. (2004) performed a study in Washington and reported that reading magazines related to appearance had an indirect impact on dissatisfaction with body image through internalization of media-communicated ideals (11). SATAQ-3 is one of the most used tools in the field of socio-cultural attitude towards appearance that has been used in different studies (12, 13). Because of the importance of this questionnaire to survey body image, psychometric studies on this questionnaire were conducted in different countries (14-16). Considering the fact that each society cultural context grows and emphasizes its specific values, it is essential to pay attention to these concerns and their consequences in Iran’s cultural context, particularly among the individuals who are more prone to such attitudes and behaviors. In this regard, SATAQ-3 was selected due to investigation of various aspects of sociocultural effects on individuals’ attitude towards their appearance, easiness of use, and conformity of items to Iranian culture. This study aimed to determine the psychometric characteristics of SATAQ-3 for accurate assessment of body image among Iranian female adolescents.

## Materials and Methods

This methodological study was conducted in Shiraz University of Medical Sciences, South of Iran, from May to October 2015. Psychometrics of the used instrument were assessed among female adolescents in Shiraz. After explaining the study objectives to the participants and reassuring them about the confidentiality of their information, their written informed consents were obtained. 

***Instrument***

Thompson et al. developed the self-report SATAQ-3 in 2004 after modifying of SATAQ and adding some items related to the effects of athletes and exercise on sociocultural factors of the societies. SATAQ-3 is one of the gold instruments used around the world (17). This questionnaire investigates various dimensions of sociocultural effects on individuals’ attitude towards their appearance. It includes 30 items, covering four issues; namely, information (media as an important source of information about appearance, 9 items), media pressure (media pressure for having an ideal appearance, 7 items), internalization-general (9 items), and internalization-athlete (5 items). These items are scored based on a 5-point Likert scale ranging from 1 (completely disagree) to 5 (completely agree) (17). Thompson et al. recommended utilization of SATAQ-3 because of its great psychometric properties, the availability of its translated versions in different languages, and its structure dimensions (18).

***Procedure***

***Translation***

Two bilingual experts cooperated with the researcher to translate SATAQ-3, using forward-backward method. At first, one of the experts translated the questionnaire into Persian. Then, the other expert back translated it into English. Finally, both experts and a clinical psychologist compared the two versions and presented the Persian version of SATAQ-3.

***Face Validity***

At this stage, for face validity, using convenience sampling, the questionnaire was given to 25adolescents. The criteria for inclusion were as follows: Female gender, age range of 15-19 years, and willing to participate in the study. In addition, the questionnaire was given to a psychologist and three nurses, who were familiar with the concept under investigation and instrumentation. Then the participants were required to evaluate the items with respect to problems, ambiguity, relativity, proper terms and grammar, and understandability using a 5-point Likert scale ranging from 1 (not important at all) to 5 (highly important)(19). Exclusion criteria included a non-return and failure to respond to all questions in the questionnaire. Then, all the questionnaires were collected and analyzed, the impact score was computed for each item, using the following formula and scores > 1.5 were considered acceptable.

Impact score = Frequency (%) × Importance (19).

***Content Validity***

Content validity involves qualitative and quantitative phases, and it was assessed by those experts, who were familiar with the concept under investigation and instrumentation. In this study, convenience sampling and both qualitative and quantitative approaches were used. In the qualitative approach, 21 experts, who were familiar with psychometric methods, were considered, but only 15 of them met the criteria. The criteria were as follows: Being expert in clinical psychology or nursing, having two years of working experience in clinical wards, and being familiar with instrumentation processes.

 After all, the questionnaire was given to two individuals with PhD in psychology, eight nursing PhD candidates, three individuals with M.Sc. in nursing, and two clinical psychologists, who were required to evaluate the items with respect to appropriate wording and grammar, understandability, and relatedness to Iranian culture and to mention their suggestions, if any, next to each item. Then, the questionnaires were collected and the suggestions were considered. In the quantitative approach, on the other hand, the modified questionnaires were given back to the experts for assessment of content validity ratio (CVR). They were also required to review the items with respect to being necessary, beneficial, or unnecessary. Then, the questionnaires were collected, and CVR was calculated for each item. Thereafter, the modified questionnaires were returned to 15 experts, who were required to analyze the items concerning relativity, simplicity, and clarity, using a 4-point Likert scale ranging from 1 (the lowest) to 4 (the highest). All of the 15 questionnaires were collected, and content validity index (CVI) was computed for each item as well as for the whole questionnaire. In this study, CVI > 0.8 and CVR > 0.45 were considered to be acceptable (20).


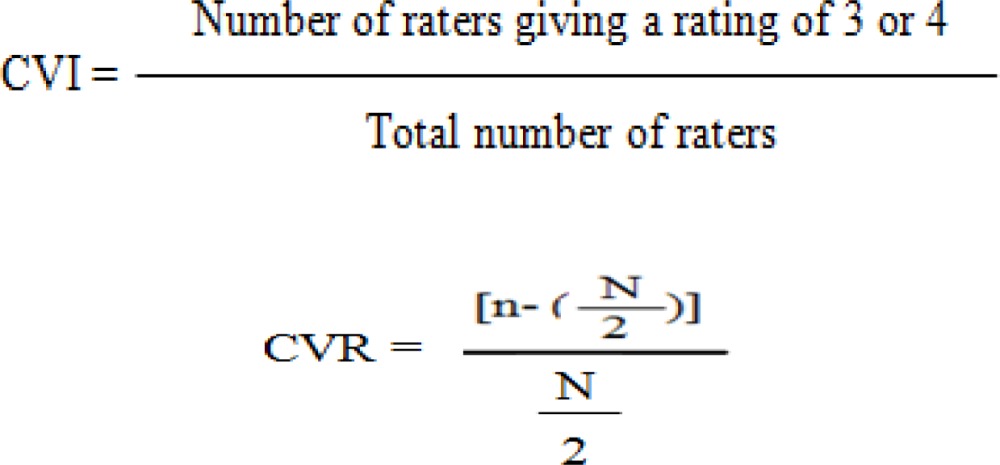


***Reliability***

Using simple random sampling method, 150 individuals with female gender, aged 15-19 years, who were willing to participate in the study were selected and invited to take part in the study. Exclusion criteria included a non-return and failure to respond to all questions in the questionnaire. 

 The reliability of the questionnaire was assessed, using internal consistency. Cronbach’s alpha coefficients > 0.7 represented acceptable reliability (21).

## Results

***Face Validity***

At this stage, all the participants stated that all the questionnaire items were simple, clear, and related to the objectives. Additionally, the impact scores of all the items were above 1.5.

***Content Validity***

Based on the participants’ opinion, items 2, 13 and 29 required review and modification. Thus, “I’ve felt pressure from TV or magazines to lose weight”, “Magazine articles aren’t important sources of information about fashion and being attractive”, and “famous people are an important source of information about fashion and being attractive” were changed into “By watching TV and reading magazines, I have become interested in getting fit”, “Journal articles are important sources of information about fashion and attractiveness”, and “Famous individuals are important sources of information about fashion and attractiveness”, respectively. Considering the experts’ opinion about the necessity of each item, CVR was computed for the items. Based on Lawshe table, the acceptable CVR for the 15 experts was 0.45 (12). In this study, CVR was between 0.78 and 1 for all the questionnaire items. Afterwards, CVI was calculated to be between 0.89 and 1 for all the items. Moreover, the means of CVR and CVI were, respectively, 0.81 and 0.91 for the whole questionnaire (Table 1).

***Reliability***

Internal consistency of SATAQ-3 was assessed, using Cronbach’s alpha method. Accordingly, Cronbach’s alpha was between 0.89 and 0.95 for the questionnaire’s dimensions, and the highest reliability was related to information and media pressure. The reliability of the dimensions is presented in Table 2. 

## Discussion

This study aimed to determine the psychometric properties of SATAQ-3 in female adolescents. To achieve the study goals, first, the questionnaire was translated into Persian and then, its reliability and validity were assessed. The results of content validity indicated that the items of the questionnaire were understandable and related to Iranian culture. Besides, the means of CVI, CVR, and the impact score were acceptable. The reliability of the questionnaire was estimated to be 91%. Thus, the questionnaire benefitted from acceptable face and content validity to assess body image, which is consistent with the results of other studies (16, 22 and 23). Hosseini et al. (2015), used CVI and CVR to assess psychometric property of the questionnaire in a study (24). In another study, the reliability coefficients of the SATAQ-3 were calculated, using internal consistency and spilt-half methods, which were 0.77 and 0.55, respectively (25). The population being different could be the reason for the difference in the results of the reliability.

Also, Warren et al. reported that SATAQ-3 had appropriate psychometric features for American-European, African-American, and American-Asian female students in the U.S. (22). Construct validity and reliability of SATAQ-3 have also been evaluated in various studies (15-17, 22, 23, 26 and 27). However, no studies have investigated its content and face validity. Mohammad-panah et al. only assessed the construct validity of this instrument in Iran in 2015, and demonstrated that it had four dimensions (internalization-general, information, internalization-athlete, and media pressure) with values above 1, which determined 5.18%, 6.06%, 9.01%, and 17.36% of the variance, respectively (25). Nonetheless, Swami et al. carried out a research in Malaysia to investigate body image among 554 young girls and women and reported that, on the contrary to the original version, this questionnaire showed three dimensions (16). On the other hand, Madanate et al. (2006) performed a study in Georgia and disclosed that the instrument was divided into four dimensions, which is in agreement with the original instrument (15).

Thompson et al. (2004) also evaluated the divergent construct validity of the questionnaire, and revealed a strong correlation between all the four dimensions of body image and nutritional disorders. 

Additionally, the reliability of all the four dimensions was above 0.89 (17). 

In the same line, Calogero et al. (2004) conducted a study on 440 women with nutritional disorders, and reported that the reliability of all the four dimensions of SATAQ-3 was above 0.9, indicating its high internal consistency (14).

**Table1:** Content Validity Ratios and Content Validity Index of the Sociocultural Attitude towards Appearance Questionnaire-3

**Cronbach’s alpha Coefficient**	**Mean(SD)**	**Dimensions**		
**Questions**			**CVI**	**CVR**
1. I’ve felt pressure from TV and magazines to diet			1	1
2. watching TV and reading magazines, I have become interested in getting in shape			0.89	0.78
3. I’ve felt pressure from TV or magazines to have a perfect body.			0.89	0.78
4. I’ve felt pressure from TV or magazines to lose weight			0.89	1
5. I compare my appearance to the appearance of people in magazines			0.89	0.78
6. I’ve felt pressure from TV or magazines to change my appearance			1	0.78
7. I compare my body to the bodies of TV and movie stars			0.89	0.78
8. I would like my body to look like the people who are in movies			1	0.78
9. I compare my appearance to the appearance of TV and movie stars			0.89	0.78
10. I would like my body to look like the models who appear in magazines			1	1
11. I’ve felt pressure from TV or magazines to exercise.			0.89	0.78
12. I wish I looked like the models in music videos			0.89	0.78
13. I compare my body to the bodies of people who appear in magazines			1	0.78
14. I’ve felt pressure from TV and magazines to look pretty			1	0.78
15. Magazines advertisements are an important source of information about fashion and ‘being attractive’.			0.89	0.78
16. Movies are an important source of information about fashion and ‘being attractive			1	1
17. Pictures in magazines are an important source of information about fashion and ‘being attractive			0.89	1
18. TV commercials are an important source of information about fashion and ‘being attractive			0.89	0.78
19. Famous individuals are important sources of information about fashion and attractiveness			0.89	0.78
20. TV programs are an important source of information about fashion and ‘being attractive			0.89	1
21. Journal articles are important sources of information about fashion and attractiveness			0.89	0.78
22. Movie stars are an important source of information about fashion and ‘being attractive			0.89	0.78
23. Music videos on TV and an important source of information about fashion and ‘being attractive			0.89	0.78
24. I compare my body to that of people who are athletic			0.89	0.78
25. I try to look like sports athletes			0.89	0.78
26. I wish I looked as athletic as sports stars			0.89	0.78
27. I compare my body to that of people in ‘good shape			1	1
28. I wish I looked as athletic as the people in magazines			0.89	0.78
29. I try to look like the people on TV			1	0.78
30. I would like my body to look like people who are on TV.			1	0.78

**Table2:** Mean, Standard Deviation, and Cronbach’s Alpha of the Sociocultural Attitude towards Appearance Questionnaire

**Cronbach’s alpha Coefficient**	**Mean(SD)**	**Dimensions**
information	42.21(3.28)	0.95
Media pressure	33.48(2.41)	0.92
internalization-general	32.67(1.89)	0.90
internalization-athlete	21.34(3.26)	0.89
total	32.42(18.94)	0.91

## Limitations

Since the study participants were selected from two high schools, the results might not be generalizable to other populations. Therefore, future multicenter studies with larger sample sizes are recommended to be conducted on this issue. Another limitation of this study was its lack of test-retest reliability, so it is recommended that test-retest reliability be done in future studies.

## Conclusion

This version of SATAQ-3 appears to be a valid and reliable instrument in socio-cultural background of Iranian population, and can be a useful tool for conducting research in the future.
